# Quackery in High Places

**Published:** 1845-06

**Authors:** William W. Dwinelle

**Affiliations:** Fellow of the American Society of Dental Surgeons.


### ARTICLE II.

*Quackery in High Places.* By WILLIAM W. DWINELLE, Fellow of the American Society of Dental Surgeons.

PERHAPS there is nothing in the whole range of the honorable and scientific dentist's experience, more calculated to mortify and confound, and call forth his most sincere regret, than the abuse of his profession by those "high in authority," those who have been elevated by some strange freak of fortune to a high station, which they are either too dishonest or too ignorant to maintain with honor to themselves or credit to the profession.

Ordinary quackery, in all its variety, presents nothing so formidable as this; the one is already withering under the scorching rays of the light of science and truth, and will, ere long, pass as the chaff before the wind; while the other will thrive, in a more secret and deeper channel, cloaked over, and covered as it is by a, perhaps, once merited reputation. Unfortunately for those better spirits, who would give the profession a more exalted character, and strip it of the imperfections and abuses that hang around it, the public are too willing to believe, that a "good business" is conclusive evidence of "good works;" that crowded operating rooms bespeak talent, and pathological research, on the part of the operator; and, above all, that if an individual has once gained and merited a reputation, we have no right to question it *now;* deeming it impossible for him to fall. They look from a wrong point of view, and judge from *appearances* merely, without reference to facts. Quacks, of every profession, are aware of this *weakness* on the part of the public, and, hence, consider, that if they can only succeed in putting on a "goodly *outside*," their success is certain; their real merit being a matter of minor importance, and wholly unworthy of consideration, especially, as they have adopted the very liberal and commendable principle, that "the *end* justifies the means." How many there are in our profession, who, in the early part of their career, seemingly gave promise of great usefulness in the field in which they had chosen to act. Their operations were performed with the greatest care; every thing that came from their hands gave evidence of skill, of a growing skill, and above all, of an honest heart, and a sincere desire to advance the profession. And yet, they have passed up to a point to which they were *satisfied*, and from which they have begun to retrogade; and subsequent facts justify us in the opinion, that their only object was, first, to gain a reputation, and then a fortune. Making their reputation only serve them as a hobby on which they may ride to riches. I cannot conceive a character more contemptible than the one just described; one who virtually gains by intrigue, the confidence and respect of the public, and then so wantonly trifles with his good name, forgets the liberal and noble-hearted spirit that should animate him, and makes a sacrifice of all of his manliness, and all of his dignity, at the shrine of selfishness. Not that I have the slightest objection to my brethren getting rich, and securing to themselves much of this world's goods, so long as they do it with honor to themselves and credit to the profession; and there is no doubt but that the two objects can move along together, hand in hand. Some of the most useful and brightest lights in our profession, furnish us with the most emphatic instances of this kind. But the spirit that gives up wholly to selfishness, making this the prime moving cause, the sole object and end, is the one I would deprecate. Important operations from their hands, hurried and slighted in such a manner, that you are led to doubt whether they, or their *blacksmith*, may claim the *credit* of them. Advice requiring research and investigation, and which may involve the happiness of the patient for life, *passed* by those oracles with a *word*, either because they are too indolent to be generous, or their selfishness assures them that the time spent in investigation, and doing justice to the patient, would yield a greater profit some other way. Crowding the work of a week into a day, because it is more profitable. Treating the living organs of the mouth as though they were sticks and stones, regardless of nerves, and flesh, and blood, because it will take less time. Secretly using nostrums, pastes, cements, &c., condemned by the honest and intelligent, because *they*, too, with other quacks, have learned how much *easier* it is. Extracting teeth one day, and replacing them on plate the next, because the patient, assured that it is "just as well," is in a hurry for them, and the operator is in a *hurry* for his pay. These are some of the evils that hang around the door of many of those who dwell in "high places," who have got a reputation, and are now getting a fortune.

I know men who will set half a dozen teeth on plate, fill a score or more, and do a large sprinkling of pivot-work in one day! and yet these men are most "respectable and scientific dentists"—that is, they *have* been, and by a strange dulness on the part of the good public, they retain their high station, with, perhaps, less merit than at any other period of their lives. I know the statement I have just made is a bold one, and I may be taxing credulity almost beyond endurance; but just pass behind the curtain with me, let me introduce you into the *sanctum*, and I will prove it to you. It shall be in the morning, so that there shall be plenty of *time!* Our hero has long since discarded the troublesome and vulgar practice of taking impressions from the mouth, because he rarely if ever uses swedges. A five or ten dollar piece is passed through a plating mill until it is exceedingly thin, as then it will *bend* so much easier. It is then cut and bent into *shape* with forceps and pliers until it "fits" to his satisfaction; clasps are hurriedly adjusted; an *idea* as to the whereabouts the teeth *ought* to be, is obtained, a scratch by a file indicate the places, linings are soldered, the teeth are *rivetted* on, and the work is done! The teeth are *stuffed*, without being stopped at all properly. The pivot work is done much in the same way; the teeth are cut off, the nerves are drilled out *part* way as far as it is intended the pivot shall come; a few strokes of the file makes a "beautiful adaptation," the work is forced into either tooth, and the work is completed two hours before supper! True, the patient may go home feeling as though they were in a harness, the face may swell, the teeth may ulcerate and come out, the jaw exfoliate, and a protracted sickness succeed, but they are, in part, prepared for all this, for they have "heard that dental operations were severe," and they are glad to escape with their lives. By some, this may be deemed a fancy sketch, but I can assure my readers it is from *real life*. I have several specimens of this kind of work in my office at this time; plates *bent* into shape, or rather *out* of shape, presenting angles as acute and irregular as you will find them on a rock broken from the mountain, yet the authors of these specimens of quackery, actually enjoy a high reputation in some of the first cities of our union.

A lady was in my office yesterday, who resides about thirty miles from the city of ——. She had nearly the amount of work to be done, as described above. Dr. ——, of that city, informed her, if she would come down to his office in the morning, she might return by the cars in the evening, with the work completed! and I have no doubt of it, judging from several specimens of his work I have seen lately, all evidently done at *railroad speed.* Dr. —— is a man of good reputation with a large majority of the community in which he resides. But this is only one instance of scores of others that might be mentioned. The experience of almost every high-minded dentist, will refer them, with regret, to individuals of this character; who, instead of aiming at perfection, and at performing their work thoroughly and skilfully, seem only anxious to obtain money, totally regardless of all consequences; who, instead of adopting the principle that "what is *well* done, is done quick enough," assume that what is done quick, is done well enough! In cases like these, not the least aggravating, is the fact, that these same individuals are *capable* of doing better, and are actually competent to perform skilful and commendable operations.

Perhaps some of my readers may think I have used the terms "distinguished" and "good reputation," altogether too carelessly, and in a manner that facts would not justify. But I can assure them I have not spoken at random.

A few weeks since I was occupied, for several days, in refilling with gold, for a young gentleman, a large number of cavities that were originally stopped by one of the most distinguished dentists of Europe—*distinguished,* I say; were I to mention his name, the fact would not be questioned; he is one whom we are proud to claim as an honorary member of our association. And yet, from this specimen, as well as several others from the same hand, all recently performed, I have no hesitation in saying, his operations are only on a par with those of the merest "quack dentists," who travel from house to house. Many of the stoppings had come out, and what remained showed *how* they *all* were done. They seemed to have been merely stuffed, and that, too, in the most careless manner. The cavities were all large and difficult; and when my patient informed me that they were cleaned and filled at the rate of six to eight per hour, either of which I considered myself fortunate in securing in that time, I was involuntarily reminded of the New York Dentists' Report of the "great" Mons. Mallan, the "pound-cake," &c.

A few months since, I had occasion to repair the teeth of a young officer in the army, who had been spending several years in Florida. I saw in his mouth, beautiful gold stoppings, right by the side of which—scarcely less difficult to control—were corroded and blackened ones of mercurial cement, and both the work of the same hand. The author of this operation is one of the first dentists in the city of New Orleans, and, no doubt, once merited the reputation he now retains. I found, by conversation with my patient, that he had been almost constantly salivated since the operation, the periosteum of several of these teeth was destroyed, a constant discharge came from the gums, and about their necks, some of which were already loose, and exceedingly fetid breath was not the least of his misfortunes. Extraction seemed the only remedy, and I took out several large molars, some of which were adorned with a gold stopping in front, and one of *mercury* behind! I have known instances where individuals have been active in expressing, publicly, their disapprobation of the use of the "*Crawcour*," or mercurial cement, even voted on resolutions against it before a body of scientific gentlemen, and yet are, even now, secretly in the habit of using it.

These are some of the reasons for complaint and mortification, and, on the part of young practitioners, of almost discouragement; who, oftentimes, and no matter how honest their purposes, strive, in vain, to have their voices heard above those quacks who dwell in high places. Our fathers in the profession, those who are so far established as to be above influences like these, cannot tell, with what a weight of discouragement, mortification and disgust, facts like these come upon those of less years and experience.

I would not, by any means, intimate that there is any *real* cause for discouragement; though here we find a most formidable foe, I have too much confidence in truth, and the light of science, to admit that we have any right to be discouraged.

There are many and increasing influences to which the young practitioner can refer with confidence and satisfaction, feeling that he is supported at a quarter to which he is proud to refer.

Let us, before we close, contemplate this more pleasing side of the picture. Notwithstanding the abuse of our profession, we can refer to many, who, even in their prosperous days, after their profession is won, keep a keen eye to *improvement*, and the advancement of our science. Instead of being influenced by a spirit of selfishness, they make it the great study of their hearts to ameliorate the condition of mankind, and secure to the present and future generations the advantage of their research and experience; who aim at *perfection*, and encourage others to strive for the same goal.

The annual meetings of our association, and, perhaps, more particularly, our ably conducted journal, are both pouring such a flood of light over the land, on the subject of our much abused profession, that "high places," as well as the low, will soon be within the reach of its influence. Our dental library is rapidly increasing, not only through the medium of our journal, but facilities for its increase are fast opening in other quarters. We have noble spirits in our brotherhood, who have been aware of our deficiency in this respect, and have stepped forward with a generous and philanthropic purpose to fill the gap, and nobly are they doing their duty. I cannot allude to a more pleasing instance of this kind, than by referring to a second and much enlarged edition of Dr. Harris's work on "*The Principles and Practice of Dental Surgery*," recently published at Philadelphia. It is the most full and complete treatise on the subject of dental science, probably, ever published. Dr. H. has great reason to congratulate himself, that he has supplied our profession with a work so useful, and one which will always find a conspicuous place in the library of the scientific dentist. He has rendered our profession an important service, for which we cannot be too grateful. Other books of a similar character, are being put forth by those who are influenced by the laudable ambition of doing *good*, and writing their names upon the *hearts* of those who shall succeed them.

Such men will be revered to the latest time. Their names will go down to posterity associated with such worthies as Rush, Bell, Hunter and others. They may go down life's decline with confidence of this fact, and with the satisfaction that they have given our profession an impulse that will never cease to be felt.

Notwithstanding we have cause for complaint, we have none for discouragement. We have every reason to look forward with confidence to the time, when dental quackery, in all its forms, shall cease; when our professsion shall command universal respect, and be clothed in the dignity it merits.

